# Supplementary material for: Micro- and Macro-Heterogeneity of N-Glycoproteome Remodeling in Endoplasmic Reticulum Stress
Source: Mol Cell Proteomics. 2026 Sep 1;25(10):101653. doi: 10.1016/j.mcpro.2026.101653 (PMC13639171; doi:10.1016/j.mcpro.2026.101653)
Supplement: Supplementary file 1 [file mmc11.docx]

**Micro- and macro- heterogeneity of N-glycoproteome remodeling in endoplasmic reticulum stress**

^1,2^Alexander Black, ^2^Tiffany Ngo, ^2^Boomathi Pandi, ^2^Andrew Mongin, ^2^Dominic CM Ng, ^2^Edward Lau, ^1,2,3^Maggie P. Y. Lam

^1^Structural Biology, Biochemistry, and Biophysics Graduate Program
^2^Department of Medicine
^3^Department of Biochemistry & Molecular Genetics
University of Colorado Anschutz Medical Campus
Aurora, CO 80045, USA

Correspondence: Maggie P. Y. Lam, PhD (maggie.lam@cuanschutz.edu)

# Supplemental Figures

## Supplemental Figure S1. Correlation of mPBA enriched glycopeptide intensities across replicates and input amounts.

Scatterplot represents MS1 intensities of glycopeptides across two biological replicates in each tested condition.

## Supplemental Figure S2. Thapsigargin and tunicamycin induce robust unfolded protein response in human AC16 cardiac cells

**A:** Heatmap of AC16 protein expression after exposure to 1 µM thapsigargin, 1 µg/mL of tunicamycin or DMSO vehicle for 24 hours. Color: log2 centered intensity; Numbers: clusters.

**B:** Comparison of the log2-fold change between conditions. The fold change with thapsigargin is displayed on the y-axis and fold change with tunicamycin is on the x-axis. Proteins are colored based on their position in the matrix. Only proteins that have a p-value less than 1 are shown outside the “unchanged” box.

**C:** Principal component analysis of protein abundance in normal, thapsigargin, and tunicamycin cells.

**D:** Gene set enrichment analysis (GSEA) results using the Reactome pathway database to evaluate the top 10 most upregulated and top 10 most downregulated pathways upon exposure to tunicamycin (top) and thapsigargin (bottom).

**E:** Dynamic range of protein LFQ intensity over protein rank in each experiment.

## Supplemental Figure S3. The effect of ER stress on the AC16 intact glycopeptide identification

**A:** Boxplots showcasing the total number total glycopeptide-spectrum matches (gPSMs) from AC16 cells after enrichment with the mPBA method. Box: IQR; whiskers: 1.5x IQR. *: P < 0.05; **: P < 0.01; ***: P < 0.005; ****: P < 0.001; ns: not significant; two-tailed t-test.

**B:** As in A, but for the number of unique glycopeptidoforms.

## Supplemental Figure S4. The effect of ER stress on the hiPSC-CM proteome and glycoproteome

**A:** GSEA dotplot results using the Reactome pathway database to evaluate the top 10 most upregulated and downregulated pathways upon exposure to 1 µM thapsigargin, 1 µg/mL of tunicamycin or DMSO vehicle for 72 hours. Size: gene count; Color: GSEA FDR-adjusted P value.

**B:** Comparison of the log2-fold change between conditions at 72 hours. The fold change with thapsigargin is displayed on the y-axis and fold change with tunicamycin is on the x-axis. Proteins are colored based on their position in the matrix.

**C:** Boxplots detailing the number of glycosites and unique glycopeptides identified in hiPSC-CM after 72 hours of treatment. Box: IQR; whiskers: 1.5x IQR; ***: P < 0.005; ****: P < 0.001; ns: not significant; two-tailed t-test.

**D:** PCA plots of each of the glycoproteomic hiPSC-CM samples using the top 320 (72 hours) most variable glycopeptidoforms.

## Supplemental Figure S5. Temporal behaviors of collagen glycopeptidoforms

**A:** Boxplots showing the behavior of select collagen glycopeptidoforms in hiPSC-CMs under 24 and 72 hours of thapsigargin and tunicamycin treatments. Boxes: IQR; whiskers 1.5x IQR; P values: limma FDR adjusted P. The 24 hours and 72 hours data are from separate batches and the experimental conditions are only compared within each time point. The time points are not normalized against each other.

## Supplemental Figure S6. N-glycosylation of nuclear annotated protein ZNF117

**A:** Screen capture of the glycoproteome database showing identified glycan classes (green: high-mannose; teal: paucimannose) at N175 site of the nuclear annotated proteins ZNF117. Bar charts show the normalized abundance (in ppm) of the glycan classes in different experiments including in HeLa and hiPSC-CM. **B:** Representative annotated gPSM of a glycoform at the N175 site.
