# Supplementary material for: Micro- and Macro-Heterogeneity of N-Glycoproteome Remodeling in Endoplasmic Reticulum Stress
Source: Mol Cell Proteomics. 2026 Sep 1;25(10):101653. doi: 10.1016/j.mcpro.2026.101653 (PMC13639171; doi:10.1016/j.mcpro.2026.101653)
Supplement: Supplementary file 2 [file mmc12.docx]

**Supplementary Information**

**Sample processing and mass spectrometry of Endo H and PNGase F experiments.**

500 µg of AC16 lysate was digested following the SP4 protocol, dried and enriched using the mPBA protocol. These samples were split into two aliquots and dried. One aliquot was resuspended in 50 µL of 1X Glycobuffer 3 (NEB) and 1 µL of Endo H (500 units) was added at 37°C overnight. The other aliquot was resuspended in 16 µL of H_2_^18^O with 200 mM sodium phosphate pH 7. N-glycans were removed using 0.5 µL of PNGase F (250 units) overnight at 37°C. Samples were desalted using C18 spin columns (Thermo Scientific) and resuspended in 7 µL of 0.1% formic acid, 3% ACN for LC-MS/MS injection.

The entire sample volume was injected via a Vanquish Neo UHPLC system (Thermo Fisher Scientific) to an Orbitrap Exploris 480 (Thermo Fisher Scientific) mass spectrometer. Deglycosylated peptides were separated on an EasySprayTM C18 analytical column (PepMap C18, 3 μm, 100 Å, 75 μm x 15 cm) heated to 50 °C, over a 120-minute gradient with a flow rate of 200 nL/min. The mobile phase consisted of solvent A (0.1% FA in 100% LCMS-grade water) and solvent B (0.1% FA in 80% LCMS-grade ACN) with a 90 minute-gradient of solvent B from 1–23% for 106.1 minutes, 23–50% for 8 minutes, 50–99% for 1 minute, and a hold at 99% for 3 minutes. The mass spectrometer operated in positive mode with MS1 scan acquired from 400–1900 m/z at 60,000 resolution in profile mode using a maximum injection time of 80 seconds with a normalized AGC target of 300% and RF lens of 50%. Precursor ions were selected following a data-dependent acquisition. Ions with charge states 2–7 and intensity greater than 3.0E4 were selected for fragmentation using a 2 m/z isolation width and accumulated for up to 220 ms with the normalized AGC target set to 400% and fragmented using stepped-HCD at 20/30/40 NCE. MS2 scans were acquired with a fixed first mass set to 100 m/z at 90,000 resolutions in profile mode. Selected ions were then placed on a dynamic exclusion list for 120 seconds. A total cycle time of 3 seconds was used.

Raw files from PNGase F-treated and Endo H-treated glycopeptides were searched separately using MSFragger v23.1 against the UniProt Swiss-Prot canonical human protein database downloaded in January 2026 with common contaminants appended by MSFragger (containing 26,819 total proteins, of which 20,420 are human protein entries). The search parameters were as follows: Digestion enzyme was trypsin cleaving at any K/R, Peptide length between 7–50 residues, up to 2 missed cleavages allowed, precursor mass tolerance 20 ppm, fragment mass tolerance at 20 ppm, maximum three variable mods (including oxidation of methionine, protein N-term acetylation). Carbamidomethylation of cysteine was assigned as a fixed modification. For Endo H-treated samples, a variable mod of single HexNAc on an asparagine was allowed. For the PNGase F treated samples, a variable mod of a single deamidated asparagine in H_2_^18^O was allowed. MS1 label-free quantification was performed using IonQuant v1.11.20 (31) with match-between-runs enabled. Peptides were filtered to 1% FDR.

The combined_modified_peptide.tsv files from FragPipe for both the PNGase F and Endo H treated samples were analyzed in R following the DEP workflow. Intact glycopeptides bearing 4+ HexNAc moieties were termed complex and those bearing 2 or 3 HexNAc moieties which consists of most hybrid and oligomannose structures were termed oligomannose.

See **Supplemental Table 9** for the glycosylation sites in our intact glycoproteomics data that were also found in the deglycosyated data (either endo H, PNGase F or both).
